# The Atlanta Society of Medicine

**Published:** 1890-01

**Authors:** 


### THE ATLANTA SOCIETY OF MEDICINE.

On the 17th inst. the Atlanta Society of Medicine held its annual meeting for the election of officers and the transaction of other routine business. Dr, Hunter P. Cooper retired from the presidency and was succeeded by Dr. Wm. Perrin Nicolson, who was unanimously chosen as presiding officer for the ensuing year.

This Society is an organization of which the profession may justly feel proud. In a quiet, unostentatious way it has accomplished a marvelous amount of good in the past few years. Its scientific work has not been without value, and many able papers have been read and many valuable discussions have taken place. But its best work has been in another direction, and the results accomplished are noticeable in the highest degree. By the frequent and intimate association of its members, and by the exclusion from its meetings of all disagreements and differences which may exist outside of its walls, it has been the means of so obliterating animosities, jealousies and petty rivalries, that it can truly be said that the profession of Atlanta is to-day harmonious and united to a degree which is probably not equaled by any other city of its size in America. That this has not always been true will be readily admitted by any one conversant with the facts. That this fortunate state of affairs is the direct outgrowth of the work of the Atlanta Society of Medicine is equally evident to any discerning mind.

The past year has been the most prosperous in the history of the Society. The meetings have been well attended, the membership has increased and the proceedings have been valuable and entertaining. A large portion of this prosperity is directly due to the efficient manner in which Dr. Cooper has filled the presidential chair. By his faithful and well-directed efforts he has elevated the Society to a higher plane than it has ever before occupied. The regret which is felt in the loss of his services in this capacity is mitigated only by the wisdom shown in the choice of his successor.

There is every reason to believe that a glorious future lies before this organization. By the avoidance of the rocks on which previous societies have split it has reached its present height of usefulness and success. The continuation of the same policy in the future warrants the expectation that it will remain a potent instrument for good, not only in stimulating good professional and scientific work, but in maintaining a high standard of harmony and unity among its members, and in promoting that good fellowship and *esprit du corps* which render all codes of ethics superfluous and unnecessary.

